# An active learning machine technique based prediction of cardiovascular heart disease from UCI-repository database

**DOI:** 10.1038/s41598-023-40717-1

**Published:** 2023-08-21

**Authors:** Saravanan Srinivasan, Subathra Gunasekaran, Sandeep Kumar Mathivanan, Benjula Anbu Malar M. B, Prabhu Jayagopal, Gemmachis Teshite Dalu

**Affiliations:** 1 Department of Computer Science and Engineering, Vel Tech Rangarajan Dr.Sagunthala R&D Institute of Science and Technology, Avadi, Chennai India; 2 Department of Computer Science and Engineering, Sathyabama Institute of Science and Technology, Chennai, India; 3 School of Computing Science and Engineering, Galgotias University, Greater Noida, 203201 India; 4 School of Computer Science Engineering and Information Systems, Vellore Institute of Technology, Vellore, 632014 Tamil Nadu India; 5 Department of Software Engineering, College of Computing and Informatics, Haramaya University, POB 138, Dire Dawa, Ethiopia

**Keywords:** Diseases, Health care, Medical research, Computational neuroscience

## Abstract

Heart disease is a significant global cause of mortality, and predicting it through clinical data analysis poses challenges. Machine learning (ML) has emerged as a valuable tool for diagnosing and predicting heart disease by analyzing healthcare data. Previous studies have extensively employed ML techniques in medical research for heart disease prediction. In this study, eight ML classifiers were utilized to identify crucial features that enhance the accuracy of heart disease prediction. Various combinations of features and well-known classification algorithms were employed to develop the prediction model. Neural network models, such as Naïve Bayes and Radial Basis Functions, were implemented, achieving accuracies of 94.78% and 90.78% respectively in heart disease prediction. Among the state-of-the-art methods for cardiovascular problem prediction, Learning Vector Quantization exhibited the highest accuracy rate of 98.7%. The motivation behind predicting Cardiovascular Heart Disease lies in its potential to save lives, improves health outcomes, and allocates healthcare resources efficiently. The key contributions encompass early intervention, personalized medicine, technological advancements, the impact on public health, and ongoing research, all of which collectively work toward reducing the burden of CHD on both individual patients and society as a whole.

## Introduction

The healthcare industry generates a lot of data about patients, illnesses, and diagnoses, but it isn't being used correctly to produce the desired results. Heart disease and stroke are two of the main causes of death. According to a WHO report, cardiovascular diseases directly kill more than 17.8 million people every year. Because there isn't enough analysis, the healthcare industry's huge amounts of patient, illness, and diagnosis data don't have the effect on patient health that was hoped for^1^. Heart and blood vessel diseases, or CVDs, include coronary artery disease, myocarditis, vascular disease, and other conditions. Stroke and heart disease kill 80% of all people who die from CVD. Three-quarters of all people who die are under the age of 70. The main things that put you at risk for cardiovascular disease are your gender, smoking, age, family history, poor diet, lipids, lack of physical activity, high blood pressure, weight gain, and drinking alcohol^2^. High blood pressure and diabetes are two examples of things that can be passed down and make you more likely to get cardiovascular disease. Some of the other things that raise the risk are being inactive, being overweight, not eating well, having back, neck, and shoulder pain, being very tired, and having a fast heartbeat. Most people have chest pain, shoulder pain, arm pain, shortness of breath, and a general sense of weakness. As it has been for a long time, chest pain is the most common sign that the heart isn't getting enough blood^3^. This kind of chest pain is called angina in medicine. Some tests, like X-rays, Magnetic Resonance Imaging (MRI), and angiography, may help figure out what is wrong. On the other hand, sometimes important medical equipment is not easily accessible, which limits what can be done in an emergency. When it comes to figuring out what's wrong with your heart and treating it, every second counts^4^. Heart disease diagnostics aren't as good as they could be, and there is a huge need for better big-data analysis in cardiovascular system redesign and patient outcomes. But noise, incompleteness, and irregularities in the data make it hard to draw clear, accurate, and well-grounded conclusions from them. Because of recent improvements in technologies like big data, information storage, and retrieval, computerised intelligence plays an important role in cardiology. In order to draw conclusions from the data mined with different ML models, researchers used pre-processing techniques^5^. Using a common set of algorithms and their variations, which are used to keep track of hereditary cardiac disorders and healthy controls, it is possible to predict when the first stage of heart failure will start. Classification technique, DT, SVC, LR, and RF machines are all types of algorithms that can be used to predict cardiac arrest. When it comes to machine learning, there are three main ways to think: The three main types of machine learning are task-driven supervised ML (classification/regression), data-driven unsupervised ML (clustering), and error-driven reinforcement learning (RL). Coronary artery disease is a very common disease of the main blood vessels that bring blood to the heart muscle. Plaques, which are made up of lipoproteins, can build up in the arteries of the heart, which can lead to coronary artery disease. Atherosclerosis is the name for the buildup of these plaques^6^. Atherosclerosis slows the flow of blood through the veins to the chest and other organs. It goes up if you have heart disease, angina, or a stroke. Men and women may have different warning signs and symptoms of coronary artery disease. For example, men are more likely than women to have chest pain. In addition to chest pain, women are more likely to experience shortness of breath, nausea, and sudden exhaustion. Heart failure, chest tightness, chest pressure, and chest pain can all be signs of coronary artery disease^7^. The Heart Disease Prediction System incorporates the Naive Bayesian Classification technique to assist in making decisions. By analyzing a vast database of past heart disease cases, the system uncovers valuable insights. This model is highly efficient in identifying patients at risk of heart disease. It possesses the ability to respond to intricate queries, showcasing its strengths in terms of interpretability, access to comprehensive information, and accuracy^8^. Making accurate and timely decisions is crucial in the medical field, especially when treating patients. Machine learning (ML) techniques play a significant role in predicting diseases by leveraging the extensive data generated by the healthcare industry. In India, heart disease is a leading cause of mortality, and the World Health Organization (WHO) emphasizes the importance of timely intervention to predict and prevent strokes. This paper focuses on predicting cardiovascular disease with enhanced accuracy by employing ML techniques such as Decision Tree and Naïve Bayes, in conjunction with risk factors. The dataset utilized in this study is the Heart Failure Dataset, which comprises 13 attributes^9^. The author investigated how well two algorithms, Support Vector Machine (SVM) and Naive Bayes, performed in predicting the occurrence of heart disease and the survival status of patients. The algorithms were applied to a dataset that included sixteen attributes from the University of California, Irvine's Centre for Machine Learning and Intelligent Systems. To assess the models' performance, a confusion matrix was used to visualize metrics like accuracy, recall, precision, and error. Additionally, statistical analysis was carried out by utilizing the receiver operating characteristic (ROC) curve and calculating the area under the curve to demonstrate the accuracy of the models^10^. In this research paper, a system is introduced that employs a radial basis function neural network to accurately predict eight different types of cardiac arrhythmias. The primary focus of the study is the analysis of heart rate time series data, and the proposed algorithm is specifically designed to predict specific arrhythmias, namely Left bundle branch block, Atrial fibrillation, Normal Sinus Rhythm, Right bundle branch block, Sinus bradycardia, Atrial flutter, Premature Ventricular Contraction, and Second-degree block. The heart rate time series data utilized in the study is sourced from the MIT-BIH arrhythmia database. Both linear and nonlinear features are extracted from the heart rate time series of each individual arrhythmia. Training of the radial basis function neural network (RBFN) is conducted using 70% of the feature datasets, while the remaining 30% is dedicated to predicting the occurrence of the eight cardiac diseases. The proposed approach demonstrates an impressive overall prediction accuracy of 96.33%, surpassing the performance of existing methods documented in the literature^11^. A novel method known as Radial Basis Classification is introduced for the classification of heart disease using clinical databases. Conventional classifiers that involve multiple attributes tend to have a large number of parameters, making it difficult to determine the ideal attributes. To address this, the concept of Multivariate Function Classifier Ideas is proposed, aiming to encourage a more cohesive stochastic trend and minimize the likelihood of errors or unforeseen results. This formula proves beneficial for arranging multidimensional data and enhancing the accuracy of grouping in the analysis phase. The results of the study indicate that the suggested calculation method offers higher precision compared to previous approaches^12^. The backpropagation neural network has demonstrated satisfactory performance in predicting accuracy. However, to further enhance accuracy and determine the specific type of heart disease, the paper integrates the CBR technique with the ANN. By leveraging historical patient records, a level of accuracy reaching 97% is attained. This research not only utilizes CBR to enhance accuracy but also to predict the type of heart disease. The CBR output encompasses both the identified type of heart disease and the recommended medication. This enables a comparison between the original medication and the medication suggested by the RBF (Radial Basis Function). The medication prescribed using this approach exhibits a comparative accuracy of 98%^13^. Symptoms include trouble breathing, pain in the upper back, neck, jaw, or throat, and pain, numbness, weakness, or a chill in the limbs. Due to the narrowing of blood vessels in certain parts of the body, it is possible to have coronary artery disease and not know it until you have a heart attack, angina, stroke, or heart failure. Keep an eye out for signs of heart problems, and if you're worried, talk to your doctor. If you get checked out often, heart (cardiovascular) disease may be found earlier^14^. This proposed method uses supervised ML classifiers to show how different models can predict the presence of cardiovascular disease and evaluate the performance of these classifiers, such as the random forest, decision tree, support vector machine, XGBoost, radial basis function, k-nearest neighbour, naïve bayes and learning vector quantization.

The goal of predicting Cardiovascular Heart Disease is to develop accurate and reliable models that can assess an individual's risk of developing various cardiovascular conditions, enabling early intervention, personalized treatment, and ultimately reducing the burden of heart disease on public health.

The remaining sections of the paper are structured as follows: Section "Literature overview" provides a comprehensive review of the relevant literature. Section "Proposed methodology" presents the proposed methodology in detail. The experimental results are analyzed and discussed in Section "Experimental results and discussion". Section "Conclusion" presents the conclusion of the study, while Section "Future work" outlines future work and potential research directions.

## Literature overview

Heart rate variability (HRV) has emerged as a reliable predictor for congestive heart failure (CHF). However, challenges remain in effectively extracting temporal features and efficiently classifying high-dimensional HRV representations. To address these challenges, this study proposes an ensemble method that utilizes short-term HRV data and deep neural networks for CHF detection. The research incorporates five publicly available databases: BIDMC CHF database (BIDMC-CHF), CHF RR interval database (CHF-RR), MIT-BIH normal sinus rhythm (NSR) database, fantasia database (FD), and NSR RR interval database (NSR-RR). Three different lengths of RR segments (N = 500, 1000, and 2000) are employed to evaluate the proposed method. Initially, expert features are extracted from the RR intervals (RRIs). Subsequently, a network based on long short-term memory-convolutional neural networks is constructed to automatically extract deep-learning (DL) features. Finally, an ensemble classifier is used to detect CHF using the aforementioned features. Blindfold validation is conducted on three CHF subjects and three normal subjects, resulting in accuracies of 99.85%, 99.41%, and 99.17% for N = 500, 1000, and 2000 length RRIs, respectively, utilizing the BIDMC-CHF, NSR, and FD databases^15^. In this publication, there is a summary of past studies and an analysis of how well the algorithm works. Before training and testing different algorithms, the suggested architecture processes the data that comes in first. The author suggests using Adaboost because it makes every ML method look better. Also, the author agreed that settings could be fine-tuned to improve accuracy. Researchers came up with a deep learning strategy for analysing and spotting cardiac conditions by using the UCI dataset. They went on to say that deep neural networks could help improve the analysis and diagnosis of cardiovascular disease as a whole. Compared to other ways to improve model performance, they found that the Talos Hyper process worked the best^16^. The KNN, RF, SVM, and DT algorithms were studied as ML models for predicting heart disease with high accuracy, high recall, and high precision. As shown in their estimation method for cardiac disorders, which is hosted on the UCI ML library, SVM-based categorization was the most accurate. We looked at the results of four machine learning techniques and one neural network (NN) for spotting heart disease. This study compared algorithms for predicting cardiac dose based on things like reliability, recall, accuracy, and F1. The Deep NN algorithm was able to spot heart problems 98% of the time. In order to show that the algorithm is useful for predicting illness, they focused on how it could be used with a medical dataset. The researchers came to the conclusion that boosting and bagging are good ways to improve the performance of classifiers that aren't very good at predicting the risk of heart disease. The results showed that the accuracy of predictions went up a lot after feature selection was used, which improved the procedure^17^. Ensemble approaches were used to improve the accuracy of bad classifiers by no more than 7%. In recent years, ML algorithms have gotten a lot of praise for how accurate and useful they have become at making predictions. It is critical to be able to create and recommend models with the greatest accuracy and efficiency possible^18^. Since hybrid models use many ML techniques and data systems, they may be able to accurately predict health problems. Weedy classifiers worked better when they used bagging and boosting, and their ability to predict cardiovascular disease risk was rated well when they worked together. They made the hybrid model by using majority voting with the Bayes Net, NB, C4.5, MLP, and RF classifiers^19^. With 85.48 percent of the time, the model that was made is right. In addition to learning models, the UCI cardiovascular disease dataset has recently been used with ML methods like RF and SVM. Accuracy went up when a lot of classifiers were added to the voting-based model^20^. Based on the data, using the weak classifiers led to an increase of 2.1% in accuracy. We used ML classification methods to figure out how people with long-term conditions would do. They found that the Hoeffding classifier can predict coronary disease with an accuracy of 88.56 percent. Overall, they found that when the hybrid model was used with the desired features, it was 87.41% accurate. We used an SVM model and the Fisher score method to choose features based on the mean^21^.

We used a lot of different classification methods and feature sets to make this one-of-a-kind prediction model. The proposed HRFLM used an ANN with a deep network and 13 clinical features as inputs. Data mining techniques like DT, SVM, NN, and KNN were also looked into. Researchers have found that it's helpful to use SVM to predict who will get sick. There was a new method called "vote," and a hybrid method that combines LR and NB was talked about. The HRFLM strategy worked out to be 88.7% effective^22^. We were able to make a model to predict death from cardiac failure that takes into account a wider range of risk factors by improving the random survival forest^23^. The IRSF used a split criterion and a stop criterion that were new to the field to tell the difference between survivors and people who didn't make it. Data mining has also been used to find out if someone has a cardiovascular disease^24^. Heart diseases are still diagnosed using Bayesian, DT classifiers, NN, association law, KNN, SVM, and ML algorithms. SVM was right 99.3% of the time. Several classifiers based on machine learning have been made to predict how long a patient will live^25^. Characteristics that were linked to the most important risk factors were rated, and the results were compared to traditional bio statistical testing. Researchers came to the conclusion that serum creatinine levels and ejection fraction are the two most important things to look at when trying to make accurate predictions^26^. The ML algorithm was used to make a model for finding CVD. In this study, we cleaned and looked at the data in four different ways. The DT and RF methods got an accuracy rate of 99.83%, while the SVM and KNN methods only got accuracy rates of 85.32% and 84.49%, respectively. Using the ensemble method, another study predicted CHF by looking at HRV and using deep neural networks to fill in knowledge gaps in unrelated areas. Overall, the method suggested was 99.85% right. In a recent publication^27^, different types of data were used to make an intelligence framework. These were principal component analyses and RF-based MLA. The FAMD was applied to RF in order to value the relevant properties and predict illness. The suggested method is correct 93.44% of the time, sensitive 89.28% of the time, and specific 96.74% of the time. In order to test their theory, the authors used a set of 303 cases that were made by adding to the Cleveland dataset. In tests, the suggested DT algorithm did 75.5% better than the baseline algorithm. Heart disease is often referred to as "cardiovascular disease"^28^. Several researchers are trying to make it easier to tell if someone has heart disease. Their research on heart disease covers a lot of ground. The author used data from the Hungarian and Statlog sets to classify CVD using the reduced error pruning tree (REP tree), R tree, M5P tree, logistic regression (LR), J48, naive bayes (NB), and JRIP. People use random forest (RF), decision tree (DT), and linear regression (LR). Support vector machine (SVM), CART, linear discriminant analysis (LDA), gradient boosting (XGB), and random forest (RF) are all used^29^. The goal of this study is to find a way to figure out how likely someone is to get heart disease. The results show that SVM does better than LR because it gets 96% accuracy while LR only gets 92% accuracy. The author says that the DT model always does better than the NB model and the SVM model. SVM has been shown to be 87% accurate, DT to be 90% accurate, and LR to be the most accurate at predicting when heart disease will happen, compared to DT, SVM, NB, and k-nearest neighbour (KNN). Table 1, represents the overall performance metric comparison of state-of-the-art methods.

**Table 1:** Literature review state-of-art method (metric comparison).

Year	Author Name	Online Database	Classification Type	Performance Metric	Accuracy
2022	^20^	IoT based data	K-NN, DT, RF, MLP, NB, L-SVM	Accuracy, sensitivity, F1 score	96.12
2022	^21^	Di-ScRi database	Evimp functions, Multivariate adaptive regression	Accuracy, Specificity, Sensitivity, F1 score	91.2
2022	^22^	Hungarian-Statlog database	LR, NB, RF REP, M5P Tree, J48, JRIP	RMSE, MAE	89.7
2022	^23^	UCI repository	KNN, DT, LR, NB, SVM	Accuracy, Sensitivity, F1-Score, Specificity	93.23
2022	^24^	Congenital heart disease database of 3910 Singleton	RF-fetal echocardiography	RMSE, MAE	95.02
2022	^25^	Pathogen, Host feature	LR, KNN, SVM, RF	Accuracy, sensitivity, F1 score	94.08
2022	^26^	Heart Disease (Kaggle Repository)	KNN, RF, ANN, Ada, GBA	RMSE, MAE	90.91
2021	^27^	Heart Cleveland (UCI repository)	LR, DT, RF, SVM, HRFLM	Accuracy, Sensitivity, F1-Score, Specificity	96.22
2021	^28^	UCI Cleveland database	RF, DT, LR	Accuracy, sensitivity, F1 score	94.21
2021	^29^	UCI repository	SVM, NB, DT	Sensitivity, accuracy	94.11

The RF-based method is 97% accurate at predicting congenital heart disease, with a specificity of 88% and a sensitivity of 85%. They were able to find CVD with 94% accuracy, 95% specificity, and 93% sensitivity by using LR, MARS, EVF, and CART-ML. RF was used to predict drug targets in host-host and host–pathogen interactions related to CVD caused by microorganisms. Several ensembles and hybrid representations have been put forward to solve the problem of predicting heart disease. Based on the suggested method^30^, CVD from the Mendeley Institute, the Cleveland datasets, and the IEEE Port are all processed with a high level of accuracy (96%, 88.24%, and 93%, respectively). The author put together the LR and RF algorithms to predict heart disease and got an accuracy of 88.7%. In this study, researchers want to find out more about how calcium in the coronary arteries and plaque in the carotid arteries are related. Both are linked to a higher risk of heart disease, but they may not be causing any symptoms yet. Machine learning and the internet of things are often used to predict and diagnose illnesses right now. The author was able to predict heart problems 94% of the time with the help of mobile devices and the deep learning method. The author employs machine learning classifiers and the Internet of Things to predict heart infections before they occur^31^. At the end of the day, we want to show that ML could be a good way to solve the problem at hand. We can use ML to look at cases related to illnesses and health problems by looking at hundreds of healthcare datasets. Researchers have worked on sophisticated computer perception for reliable healthcare to find out how machine vision practises help human needs, such as psychosocial health, specific movement, exposure-induced fatigue, frequently having to watch live actions, image analysis, deep learning, pattern classification, and how language understanding and computer animation work with robotics^32^. The authors noticed and wrote about how users learn about sharp interfaces and virtual reality tools, which leads to the development of complex restorative systems that can do human activities and recognise them. The work backs up the direct method of machine vision in the healthcare sector. This includes the technology behind intelligent wheelchairs, possible help for the visually impaired, and other object tracking solutions that have recently been used to monitor health and safety^33^. Scientists used support vector machines, generalised boosting machines, logistic regression, light boosting machines, and random forests to see how likely someone was to get cardiovascular disease. RF was the best way to predict who would get heart disease. It was right 88% of the time. Our method is put up against the current study. This is the first and only study to compare the accuracy of seven different ML classifiers for predicting cardiovascular illness. These methods include the most cutting-edge ones like learning vector quantization, RBF neural networks, and logistic regression. So, it is now possible to use a system that is both accurate and useful for predicting heart problems. Also, we suggest using the best machine learning classifier when making smart systems for predicting CHD^34^. The key features of cardiovascular illnesses include high morbidity, disability, and death, and the etiology of heart disease remains an unresolved worldwide issue. Therefore, accurate early prediction of anticipated outcomes in individuals affected by cardiac illness is necessary. In this work, we employed ML modelling to predict cardiac disease. This study focuses on predicting heart disease using ML classifiers. The authors first address the dataset problem, and subsequently enhance and standardize it for tokenization and lowercase conversion. The datasets were then utilized to train and test the classifiers, assessing their performance to achieve the highest level of accuracy. These algorithms must meet strict admission criteria, including modernity, representativeness, and high maturity. Previously, we employed Naive Bayes and Radial Basis Functions by examining the works of prior researchers. We investigated whether these approaches had been utilized on the UCI heart dataset by earlier researchers.

The proposed work contributions:

i. The authors commence by discussing datasets, which are subsequently standardized and enhanced. These datasets are then employed to train and test several classifiers to determine the one with the highest accuracy.
ii. Subsequently, the authors utilize the correlation matrix to classify the optimal values or features.
iii. The third step involves applying the ML classifiers to the pre-processed dataset, aiming for the highest achievable accuracy through parameter modifications.
iv. In the fourth and final step, the suggested classifiers are assessed for accuracy, precision (specificity), recall (sensitivity), and F-Measure.

Ultimately, the suggested classifiers outperform the state-of-the-art classifiers presented in Table 1 in terms of accuracy.

## Proposed methodology

**Figure 1:**
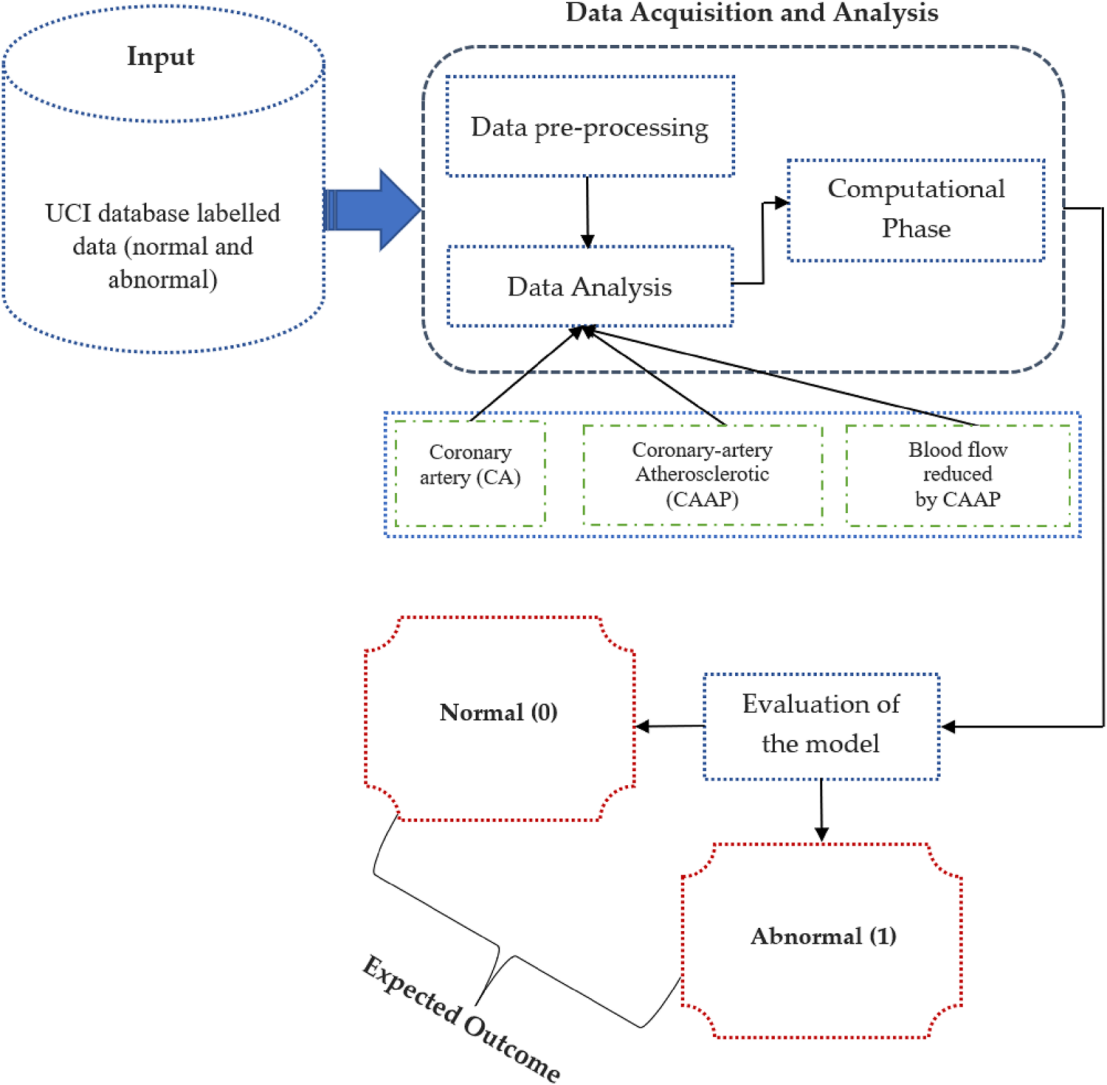
Proposed system operation overview.

With the utilization of the heart dataset, we employed ML classifiers to predict the presence of coronary heart disease. The dataset was obtained from the UCI repository, and feature engineering was applied for data pre-processing before selecting the features. Subsequently, we divided it into training and test datasets, using around 70% of the total data for training and the remaining portion for testing. The training dataset is used to create a model that predicts heart disease, while the test dataset is utilized to evaluate the classifiers. Prior to transforming categorical variables into numerical values for classification, a thorough dataset analysis was conducted. The dataset was labelled as "normal" and "diseased" in Step 1. The "diseased" label indicates the presence of heart disease, while the "normal" label indicates the absence of heart disease. In Step 2, data cleaning was performed during the training phase. Data pre-processing involved handling missing values by calculating the mean due to the presence of partial and missing values. Step 3 involved data visualization using Exploratory Data Analysis (EDA) to examine relationships between various attributes. Notably, we identified that the correlation for FBS is relatively low. Moving to Step 4, ML classifiers were applied to the pre-processed dataset, and the classifiers' performance was evaluated using a variety of parameters. As previously mentioned, the dataset was split into test and training sets to respectively assess the classifiers and develop the model. The employed classifiers demonstrated varying levels of accuracy in detecting the presence of heart disease. Figure 1 illustrates the stages of our proposed working approach.

### Dataset availability

We used the publicly available cardiovascular disease data sets from the UCI database. There are 503 cases in all, with multivariate features represented by 10 attributes, and a range of integer, category, and real values. The data set is described in Table 2. Database: https://archive.ics.uci.edu/ml/datasets/heart+disease.

**Table 2:** Dataset attributes and characters.

S. no	Features	Attributes	Character
1	Gender	Sex	Category (male or female)
2	Age-group	Uses the integer value	Fundamental aspect to divide the patient history
3	Cardiac status	Non-anginal, typical angina and asymptomatic	Creates a severe chest pain
4	Blood-pressure monitoring	Floating and integer range values	Multiple organ failure may induce
5	Fat	Floating and integer range values	High-density, low-density lipoproteins, and triglycerides
6	Diabetic-average	Binary value to represent the true or false	Exceeded the recommended value blood-sugar
7	Electrocardiogram	Representation of different normal and abnormal hypertrophy	Electrocardiogram evaluation
8	Obtained pulse rate peak value	Floating and integer range values	Exceeded the recommended heart rate value
9	Angina pectoris	Binary value to represent the true or false	It can be reduced by doing exercise
10	Obsolete peak	Floating and integer range values	Relaxation is required to compete the stress

### Proposed model overview

Using the heart dataset and ML classifiers, we were able to make accurate predictions on the presence of coronary heart disease. The dataset was obtained from the UCI-repository, and material that was previously carried out was carried out prior to feature engineering being used to pick the features. We then split it up into two portions, one for training and one for testing, with the former containing typically 75% of the total data and the latter the remainder. The training dataset is used to make predictions about cardiovascular illness, while the test information is used to evaluate classifiers. Before transforming categorical variables to quantitative data for classification, we first analyse the dataset.

Phase 1: The dataset was annotated with "normal" and "abnormal" labels. Both the "healthy" and "sick" labels indicate that the respective individuals are free of any heart-related issues. Phase 2: There was some tidying up of the data that we did. Due to the partial and missing data in the dataset, we averaged the remaining values to complete the phase. Phase 3: We used exploratory data analysis to visualise the data and look for patterns in the relationships between variables. Our research showed that the association between FBS and anything else was quite modest. Phase 4: We next examined the performance of the ML classifiers on the pre-processed dataset using a variety of metrics. As was previously said, the dataset is often divided into testing and training sets, the former of which is used to assess the efficacy of the classifiers and the latter to educate the model. Classifiers used to make predictions about cardiac health have varying degrees of success. Figure 1 depicts the stages of our suggested working method.

#### Learning vector quantization: cardiovascular classification

**Figure 2:**
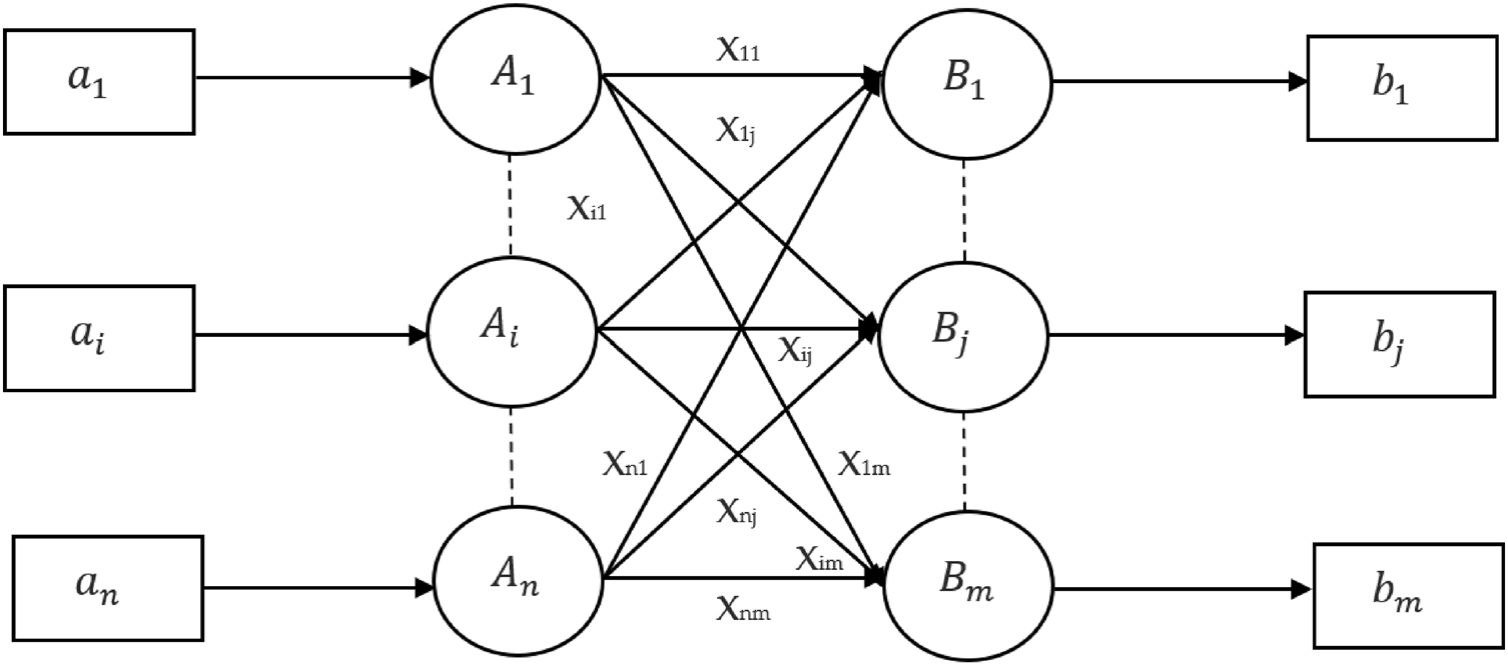
Learning Vector Quantization architecture for Cardiovascular Classification.

Learning vector quantization is a network that is based on competition and uses supervised learning. We could say that it is a method of organizing patterns into groups, in which each transfer function is a group. Since it uses a learning algorithm, the system will be given a collection of learning patterns with recognised classifications and a preliminary allocation of the output variable. After the training is done, LVQ would then categorise an input vector by placing it in the same class as the output channel. The architecture of LVQ is shown in the following Fig. 2. As we can see, there are "n" units serving as input, and "m" units serving as output. The layers are completely attached to one another and have weights placed on them. The following respective parameters have been used for LVQ training operations for cardiovascular classification:

$${\varvec{a}}\boldsymbol{ }\to {\varvec{is\, a\, suggested\, trainig\, vector\,}}({{\varvec{a}}}_{1}\dots {{\varvec{a}}}_{{\varvec{i}}}\dots {{\varvec{a}}}_{{\varvec{n}}})$$

$${{\varvec{T}}}_{{\varvec{v}}}\to {\varvec{training\, vectro\, class\, for\, }}\boldsymbol{^{\prime}}{\varvec{x}}\boldsymbol{^{\prime}}$$

$${{\varvec{W}}}_{{\varvec{j}}}\boldsymbol{ }\to {\varvec{vector\, weight\, for\, outcome\, uinit\, of\, }}\boldsymbol{{\varvec{j}}}^{{\varvec{t}}{\varvec{h}}}$$

$${{\varvec{D}}}_{{\varvec{j}}}\boldsymbol{ }\to \boldsymbol{ }{{\varvec{j}}}^{{\varvec{th}}}\boldsymbol{ }{\varvec{class\, associated\, outcome\, unit}}$$

## Algorithm 1: Learning vector quantization—cardiovascular classification

- **1. Clarification of parameters:**m (number of reference vectors): This should be specified as a parameter that determines how many reference vectors (or prototype vectors) will be used in the LVQ algorithm.
- Reference Vector β: There seems to be a confusion here. In LVQ, the reference vectors are not denoted by β. The learning rate, often denoted by or η, is the scalar that is adjusted during training.

- **2. Step-by-Step Clarification of LVQ Algorithm:**Step 1: Start
- Step 2: Initialize the reference vectors (prototypes). This involves selecting m vectors from the training set to serve as the initial reference vectors for each class.
- Step 3: Randomly assign initial classifications to the reference vectors if not done explicitly.
- Step 4: Assign the initial learning rate β (often denoted by or η).
- Step 5: Compute the squared Euclidean distance between each training vector, and each reference vector:
- Step 6: Find the reference vector Rj that is closest to the input vector (i.e., has the minimum Euclidean distance).
  1 $$ED\left({j}\right)={\sum_{{i}=1}^{n}}\sum_{{j}=1}^{m}{({a}_{i}-{X}_{{i},{j}})}^{2}$$
- Step 7: Update the reference vector based on the classification of the input vector:
- If the input vector belongs to the same class as the reference vector Rj (denoted by S=Rj), update the reference vector away from the input vector:
- This rule helps the reference vector better represent its class by moving towards the input vector from the same class.
- If the input vector belongs to the different class than the reference vector Rj (denoted by S≠Rj), update the reference vector away from the input vector:
- This rule helps to increase the distinction between classes by moving the reference vector away from the input vector of a different class.
- Step 8: Decrease the learning rate β according to a predefined schedule. This could be a linear decay, exponential decay, or another method.
  2 $${X_j^{new}}={X_j^{old}}+\beta (\alpha-{X_j^{old}})$$
  3 $${X_j^{new}}={X_j^{old}}-\beta (\alpha-{X_j^{old}})$$
- Step 9: Check for stopping conditions. Common conditions include reaching a maximum number of iterations or when changes in the reference vectors become negligible.
- Step 10: Stop

- **3. Addressing Variables and Notation:**
- Represents the components of the reference vectors.
- Represents a training vector.
- Represents the class label of the training vector.
  Rj - Represents the j-th reference vector.
  β - Represents the learning rate.

Clear coronary arteries, coronary arteries with atheromatous lesions, and coronary arteries with reduced blood flow due to blockage are all shown by the coronary artery contour. The degree and direction of a linear connection between two quantitative variables may be described by examining their correlation. Table 3 shows the relationships between the various columns. Most columns have some correlation with the "number" variable, but "BS-F" have very little.

4 $${\widehat{{\varvec{x}}}}_{{{\varvec{a}}}_{{\varvec{i}}}}={\boldsymbol{\alpha }}_{{{\varvec{a}}}_{0}}+{\boldsymbol{\alpha }}_{{{\varvec{a}}}_{1}{{\varvec{y}}}_{1}}+{\boldsymbol{\alpha }}_{{{\varvec{a}}}_{2}{{\varvec{y}}}_{2}}+{\boldsymbol{\alpha }}_{{{\varvec{a}}}_{3}{{\varvec{y}}}_{3}}+\dots {\boldsymbol{\alpha }}_{{{\varvec{a}}}_{{\varvec{n}}}{{\varvec{y}}}_{{\varvec{n}}}}$$

**Table 3:** Correlation-matrix value.

S. no	Features	Range
1	Gender	0.31
2	Age-group	0.26
3	Cardiac status	0.41
4	Blood-pressure monitoring	0.038
5	Fat1	0.082
6	Diabetic-average	0.39
7	Electrocardiogram	0.16
8	Obtained pulse rate peak value	0.41
9	Angina pectoris	0.38
10	Obsolete peak	0.31

**Figure 3:**
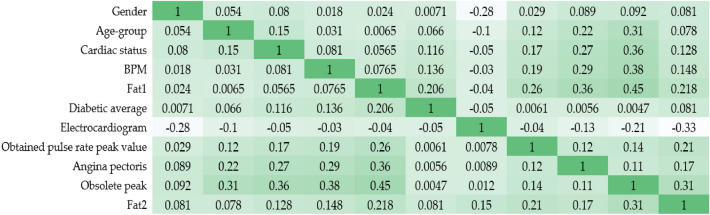
Heat map-correlation matrix.

From the Eq. (1) Correlation coefficients α between one explanatory variable (y) and another (x) are represented by a string of characters in this formula (x). The value of α1 indicates the strength of the relationship between variable (y) and independent (x) variables, and so on. Figure 3 depicts a heat map embedded inside a correlation matrix. A heatmap is a visual representation of the relationship between independent characteristics and dependent values. In addition, it is clear which characteristics have the strongest link to the supplementary characteristic’s variable. The end product is shown in Fig. 2. To better understand the data, we will now plot the characteristic of the cardiovascular disease dataset against the number. Statistic graphics and other forms of data visualisation are common tools in exploratory data analysis, which is used to examine datasets in order to identify and describe their most salient features.

5 $${\varvec{s}}=\frac{\sum \left({\varvec{a}}{{\varvec{y}}}_{{\varvec{i}}}-\overline{{\varvec{a}}{\varvec{y}} }\right)({\varvec{a}}{{\varvec{x}}}_{{\varvec{i}}}-\overline{{\varvec{a}}{\varvec{x}} })}{\sqrt{\sum {\left({\varvec{a}}{{\varvec{y}}}_{{\varvec{i}}}-\overline{{\varvec{a}}{\varvec{y}} }\right)}^{2}{({\varvec{a}}{{\varvec{x}}}_{{\varvec{i}}}-\overline{{\varvec{a}}{\varvec{x} })}}^{2}}}$$

Equation (5), the overall correlation between two variables in a sample population is given by this equation. This would be the connection between the independent variable and the dependent variable in basic linear regression. Table 4, illustrates the various age-group cardiovascular analysis.

**Table 4:** Different age-group cardiovascular analysis.

Age-group	Exemplify	Age-group	Exemplify
30	97	48	55
31	96	49	58
32	52	50	54
33	96	51	78
34	78	52	71
35	57	53	78
36	88	54	61
37	90	55	37
38	69	56	50
39	60	57	47
40	78	58	43
41	37	59	36
42	56	60	33
43	66	61	16
44	60	62	38
45	45	63	36
46	56	64	61
47	58	65	48

**Figure 4:**
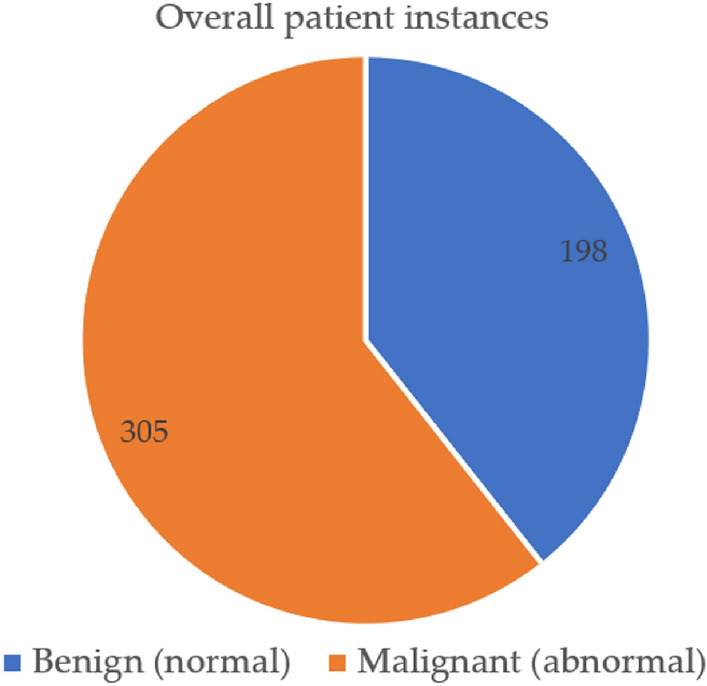
Overall heart-disease patient instances.

**Figure 5:**
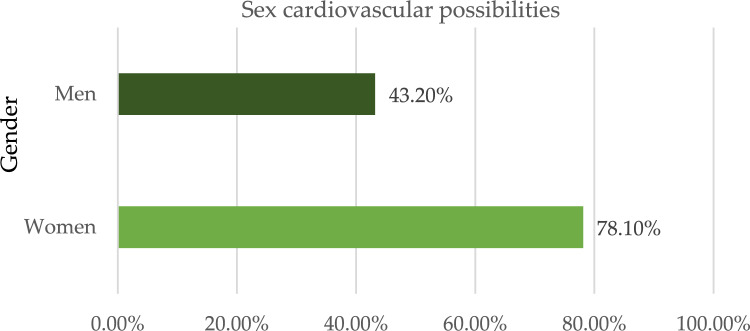
Sex categorization based cardiovascular possibilities.

**Figure 6:**
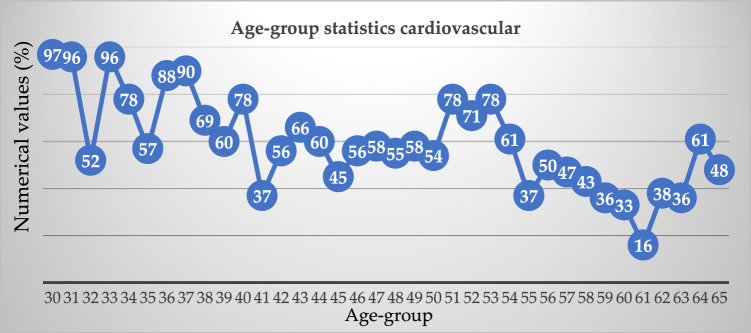
Age-group statistics cardiovascular.

Out of a maximum of 503 cases of illness, we determined that 305 individuals had some kind of heart disease issue. Malignant is represented by 1, benign by 0, and 198 of the total patients are considered healthy. Given these results, we may infer that 53.36 percent of patients have cardiac issues and that 46.64% do not. We also looked at other characteristics in the dataset, including gender, age group, cardiac status, blood pressure monitoring, fat, and smoking. Diabetic-average, the electrocardiogram obtained the pulse rate peak value for angina pectoris and the obsolete peak. As can be seen in Fig. 4, the sex property accepts two values: 0 for women and 1 for men. According to the results, women have a higher risk of developing cardiovascular disease than men do. Figure 5 shows the age distribution of the dataset, demonstrating that the risk of heart disease is independent of age group. Both age and the desired percentage are shown by the x- and y-axes, respectively. Chest discomfort is common among those who suffer from heart disease. Chest pain can be experienced by cardiovascular patients. However, the chest pain can be divided into different categories, such as non-anginal, asymptomatic, non-typical angina, and typical angina. Figure 6 depicts the different categories of chest pain that may occur. According to Fig. 6, patients with non-typical angina may have the highest risk of cardiac arrest. Blood sugar during fasting (BS-F) cannot have a significant impact on the development of heart disease.

**Figure 7:**
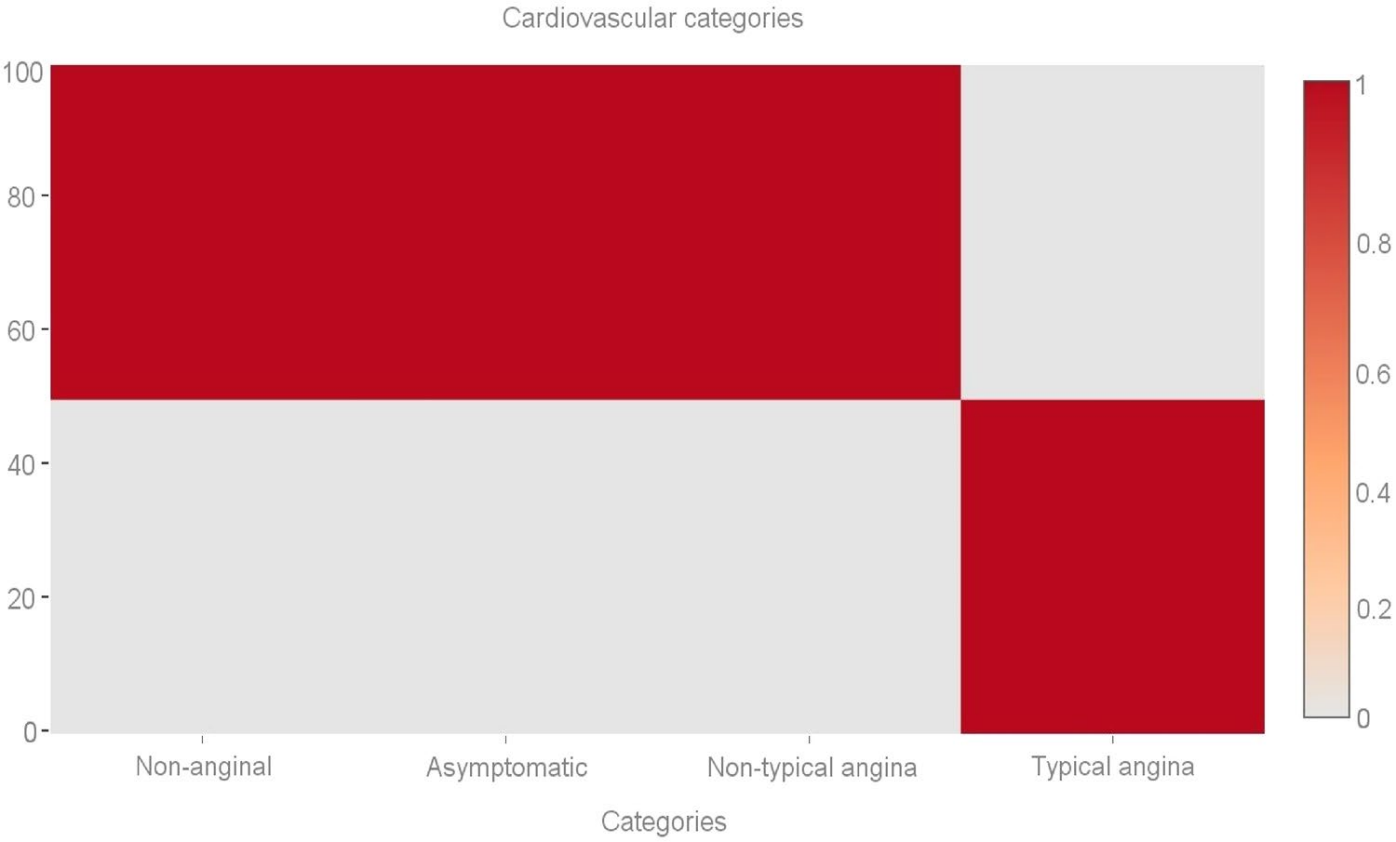
Different cardiovascular types.

**Figure 8:**
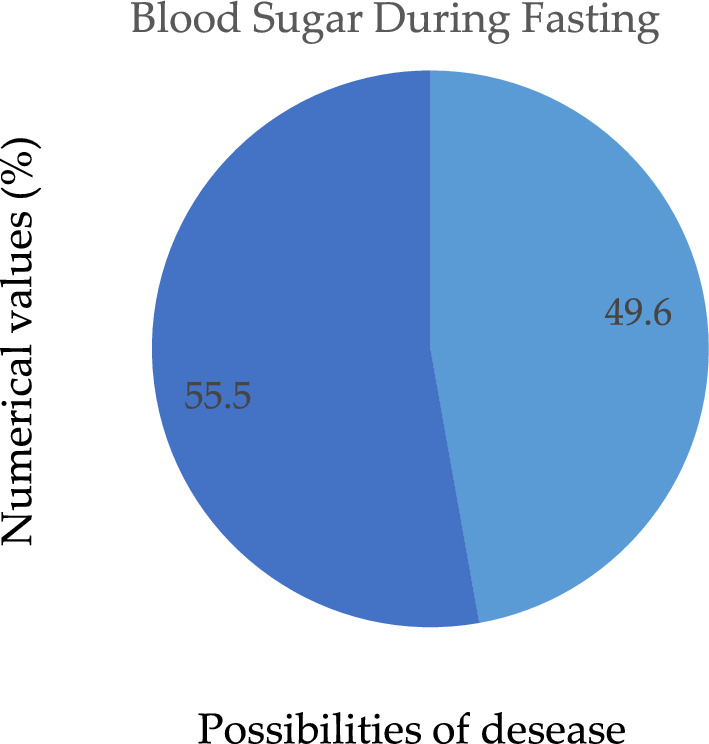
Possibility of disease during fasting.

**Figure 9:**
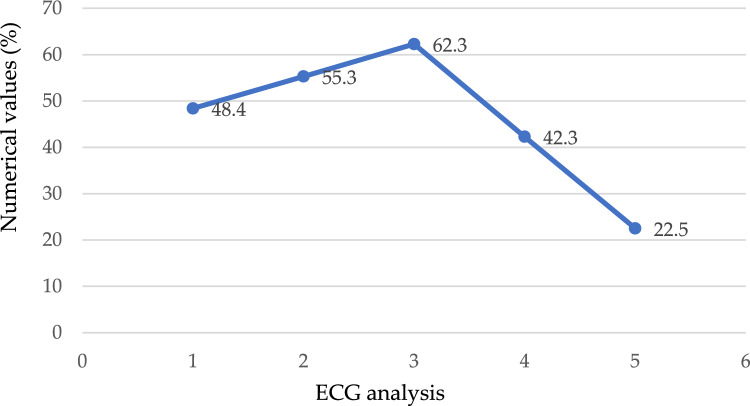
Analysis of ECG of cardiovascular possibility.

We performed an analysis on the information in which the value 1 (true) is assigned to the case in which the patient's fasting blood sugar level is more than 120 mg/dL, indicating that they are at risk for the condition; otherwise, the number 0 (false) is assigned to the case, as depicted in Fig. 7. According to the findings, there is nothing particularly remarkable about this method for predicting the existence of heart disease. Electrocardiogram readings are 0, 1, and 2. The results demonstrate that those whose ECG values are "1" or "0" have an increased risk of developing heart disease in comparison to people whose ECG values are "20," as seen in Fig. 8. Figure 9 depicts the ECG analysis of cardiovascular possibility. Table 5, represents the various categories of cardiovascular occurrence. Table 6, illustrates the chances of cardiovascular found from ECG scrutiny.

**Table 5:** Various categories of cardiovascular.

Category	Occurrence
Non-anginal	79
Asymptomatic	75
Non-typical angina	83
Typical-angina	32

**Table 6:** Chances of cardiovascular from ECG scrutiny.

Type	ECG analysis
A0	48.4
A(0,1)	55.3
A1	62.3
A(1,2)	42.3
A2	22.5

**Figure 10:**
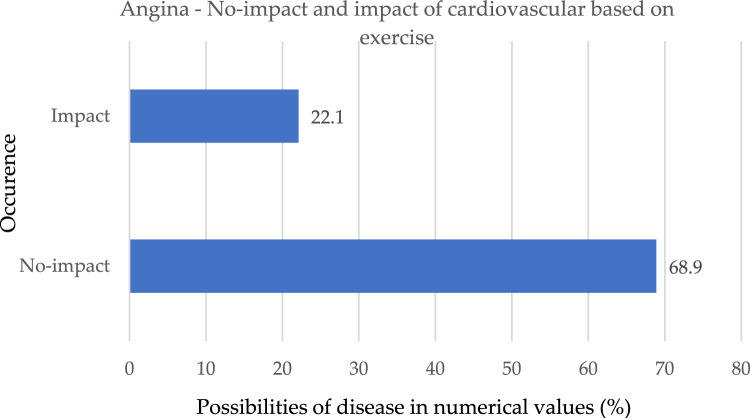
No-impact and impact of the occurrence based on exercise.

As shown in Fig. 10, those who suffer from angina have a much lower risk of developing cardiac issues. If the score of workout angina is 1, it indicates that the patient does in fact have a heart issue; on the other hand, if it is 0, it indicates that the patient does not have a heart problem and is thus less likely to develop heart problems.

## Experimental results and discussion

**Figure 11:**
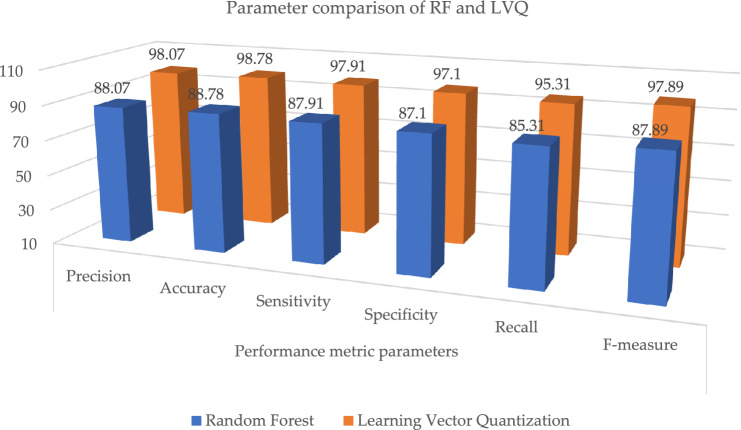
Graphical illustration of parameter metric comparison of RF and LVQ.

In this chapter, the results of ML classifiers on various evaluation requirements, such as accuracy, recall, and F-measure, are addressed. Examples of these evaluation constraints include: In addition to this, the performance of machine learning classification models is assessed using the dataset, which includes information on heart disease. k-NN did not do very well, although RBF, NB, and LVQ fared better than the other classifiers when compared to their overall performance. As can be seen in Table 7, the most important assessment criteria that were taken into consideration in this study to evaluate the performance of the ML classifier are the sensitivity, accuracy, specificity, recall, precision, and F-measure ratings. As a consequence of this, the specificity and sensitivity of the targeted class are calculated in order to evaluate the accuracy with which the given method is projected to perform. The "TP" (true positive), "TN" (true negative), "FN" (false negative), and "FP" (false positive) rates are used to compute the accuracy, precision, recall, and F measure in ML. These measures are determined by the quality of the data. Each correct positive and correct negative prediction is further subdivided into correct positive and correct negative forecasts. Every model correctly predicted the TP, TN, FP, and FN outcomes. The letters TP stand for diseased, which means infected. FN is an illness that is not believed to be related to cardiovascular disease. The FP illness is one that has been predicted but has never been seen in humans. In the actual world, TN does not exist as a disease, and this is not anticipated to change in the foreseeable future. The performance of ML approaches in terms of accuracy is listed in Table 7. By associating the performances of these classifiers, we observed that radial basis functions, naive bayes, and learning vector quantization, as well as their relatedness to other ML classifiers, led these models to achieve almost 90.06%, 94.16%, and 98.07% accuracy, respectively, as shown in Fig. 11.

**Table 7:** Parameter metric comparison of RF and LVQ.

Classification techniques	Performance metric parameters					
	Precision	Accuracy	Sensitivity	Specificity	Recall	F-measure
Random forest	88.07	88.78	87.91	87.1	85.31	87.89
**Proposed learning vector quantization**	**98.07**	**98.78**	**97.91**	**97.1**	**95.31**	**97.89**

Significant values are in bold.

6 $${\varvec{P}}{\varvec{r}}{\varvec{e}}{\varvec{c}}{\varvec{i}}{\varvec{s}}{\varvec{i}}{\varvec{o}}{\varvec{n}}=\frac{{\varvec{T}}{\varvec{r}}{\varvec{u}}{\varvec{e\,}}\boldsymbol{ }{\varvec{p}}{\varvec{o}}{\varvec{s}}{\varvec{i}}{\varvec{t}}{\varvec{i}}{\varvec{v}}{\varvec{e}}}{{\varvec{T}}{\varvec{r}}{\varvec{u}}{\varvec{e\,}}\boldsymbol{ }{\varvec{p}}{\varvec{o}}{\varvec{s}}{\varvec{i}}{\varvec{t}}{\varvec{i}}{\varvec{v}}{\varvec{e}}+{\varvec{F}}{\varvec{a}}{\varvec{l}}{\varvec{s}}{\varvec{e\,}}\boldsymbol{ }{\varvec{p}}{\varvec{o}}{\varvec{s}}{\varvec{i}}{\varvec{t}}{\varvec{i}}{\varvec{v}}{\varvec{e}}}$$

7 $${\varvec{S}}{\varvec{e}}{\varvec{n}}{\varvec{s}}{\varvec{i}}{\varvec{t}}{\varvec{i}}{\varvec{v}}{\varvec{i}}{\varvec{t}}{\varvec{y}}=\frac{{\varvec{T}}{\varvec{r}}{\varvec{u}}{\varvec{e\,}}\boldsymbol{ }{\varvec{p}}{\varvec{o}}{\varvec{s}}{\varvec{i}}{\varvec{t}}{\varvec{i}}{\varvec{v}}{\varvec{e}}}{{\varvec{T}}{\varvec{r}}{\varvec{u}}{\varvec{e\,}}\boldsymbol{ }{\varvec{p}}{\varvec{o}}{\varvec{s}}{\varvec{i}}{\varvec{t}}{\varvec{i}}{\varvec{v}}{\varvec{e}}+{\varvec{F}}{\varvec{a}}{\varvec{l}}{\varvec{s}}{\varvec{e\,}}\boldsymbol{ }{\varvec{n}}{\varvec{e}}{\varvec{g}}{\varvec{a}}{\varvec{t}}{\varvec{i}}{\varvec{v}}{\varvec{e}}}$$

8 $${\varvec{A}}{\varvec{c}}{\varvec{c}}{\varvec{u}}{\varvec{r}}{\varvec{a}}{\varvec{c}}{\varvec{y}}=\frac{{\varvec{T}}{\varvec{r}}{\varvec{u}}{\varvec{e\,}}\boldsymbol{ }{\varvec{p}}{\varvec{o}}{\varvec{s}}{\varvec{i}}{\varvec{t}}{\varvec{i}}{\varvec{v}}{\varvec{e}}+{\varvec{T}}{\varvec{r}}{\varvec{u}}{\varvec{e\,}}\boldsymbol{ }{\varvec{n}}{\varvec{e}}{\varvec{g}}{\varvec{a}}{\varvec{t}}{\varvec{i}}{\varvec{v}}{\varvec{e}}}{{\varvec{T}}{\varvec{r}}{\varvec{u}}{\varvec{e\,}}\boldsymbol{ }{\varvec{p}}{\varvec{o}}{\varvec{s}}{\varvec{i}}{\varvec{t}}{\varvec{i}}{\varvec{v}}{\varvec{e}}+{\varvec{F}}{\varvec{a}}{\varvec{l}}{\varvec{s}}{\varvec{e\,}}\boldsymbol{ }{\varvec{p}}{\varvec{o}}{\varvec{s}}{\varvec{i}}{\varvec{t}}{\varvec{i}}{\varvec{v}}{\varvec{e}}+{\varvec{T}}{\varvec{r}}{\varvec{u}}{\varvec{e\,}}\boldsymbol{ }{\varvec{n}}{\varvec{e}}{\varvec{g}}{\varvec{a}}{\varvec{t}}{\varvec{i}}{\varvec{v}}{\varvec{e}}+{\varvec{F}}{\varvec{a}}{\varvec{l}}{\varvec{s}}{\varvec{e\,}}\boldsymbol{ }{\varvec{n}}{\varvec{e}}{\varvec{g}}{\varvec{a}}{\varvec{t}}{\varvec{i}}{\varvec{v}}{\varvec{e}}}$$

9 $${\varvec{S}}{\varvec{p}}{\varvec{e}}{\varvec{c}}{\varvec{i}}{\varvec{f}}{\varvec{i}}{\varvec{c}}{\varvec{i}}{\varvec{t}}{\varvec{y}}=\frac{{\varvec{T}}{\varvec{r}}{\varvec{u}}{\varvec{e\,}}\boldsymbol{ }{\varvec{n}}{\varvec{e}}{\varvec{g}}{\varvec{a}}{\varvec{t}}{\varvec{i}}{\varvec{v}}{\varvec{e}}}{{\varvec{T}}{\varvec{r}}{\varvec{u}}{\varvec{e\,}}\boldsymbol{ }{\varvec{n}}{\varvec{e}}{\varvec{g}}{\varvec{a}}{\varvec{t}}{\varvec{i}}{\varvec{v}}{\varvec{e}}+{\varvec{F}}{\varvec{a}}{\varvec{l}}{\varvec{s}}{\varvec{e\,}}\boldsymbol{ }{\varvec{p}}{\varvec{o}}{\varvec{s}}{\varvec{i}}{\varvec{t}}{\varvec{i}}{\varvec{v}}{\varvec{e}}}$$

10 $${\varvec{R}}{\varvec{e}}{\varvec{c}}{\varvec{a}}{\varvec{l}}{\varvec{l}}=\frac{{\varvec{T}}{\varvec{r}}{\varvec{u}}{\varvec{e\,}}\boldsymbol{ }{\varvec{p}}{\varvec{o}}{\varvec{s}}{\varvec{i}}{\varvec{t}}{\varvec{i}}{\varvec{v}}{\varvec{e}}}{{\varvec{T}}{\varvec{r}}{\varvec{u}}{\varvec{e\,}}\boldsymbol{ }{\varvec{p}}{\varvec{o}}{\varvec{s}}{\varvec{i}}{\varvec{t}}{\varvec{i}}{\varvec{v}}{\varvec{e}}+{\varvec{F}}{\varvec{a}}{\varvec{l}}{\varvec{s}}{\varvec{e\,}}\boldsymbol{ }{\varvec{n}}{\varvec{e}}{\varvec{g}}{\varvec{a}}{\varvec{t}}{\varvec{i}}{\varvec{v}}{\varvec{e}}}$$

11 $${\varvec{F}}-{\varvec{m}}{\varvec{e}}{\varvec{a}}{\varvec{s}}{\varvec{u}}{\varvec{r}}{\varvec{e}}=\frac{2\boldsymbol{*}{\varvec{P}}{\varvec{r}}{\varvec{e}}{\varvec{c}}{\varvec{i}}{\varvec{s}}{\varvec{i}}{\varvec{o}}{\varvec{n}}\boldsymbol{*}{\varvec{R}}{\varvec{e}}{\varvec{c}}{\varvec{a}}{\varvec{l}}{\varvec{l}}}{{\varvec{P}}{\varvec{r}}{\varvec{e}}{\varvec{c}}{\varvec{i}}{\varvec{s}}{\varvec{i}}{\varvec{o}}{\varvec{n}}+{\varvec{R}}{\varvec{e}}{\varvec{c}}{\varvec{a}}{\varvec{l}}{\varvec{l}}}$$

Table 7 and Fig. 11, illustrates the paramenter metric (precision, accuracy, sensitivity, specificity, recall and f-measure) comparison outcome of random forest (RF) and learning vector quantization (LVQ). The outcome shows that the LVQ obtained better outcome accuracy of 98.78%. Table 8, the paramenter metric (precision, accuracy, sensitivity, specificity, recall and f-measure) comparison outcome of decision tree (DT) and learning vector quantization (LVQ).

**Table 8:** Parameter metric comparison of DT and LVQ.

Classification techniques	Performance metric parameters					
	Precision	Accuracy	Sensitivity	Specificity	Recall	F-measure
Decision tree	89.07	89.78	88.91	88.1	86.31	88.89
**Proposed learning vector quantization**	**98.07**	**98.78**	**97.91**	**97.1**	**95.31**	**97.89**

Significant values are in bold.

**Figure 12:**
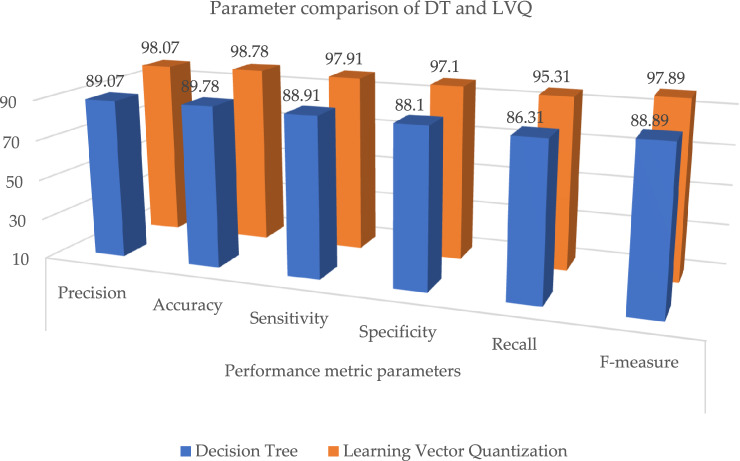
Graphical illustration of parameter metric comparison of DT and LVQ.

**Figure 13:**
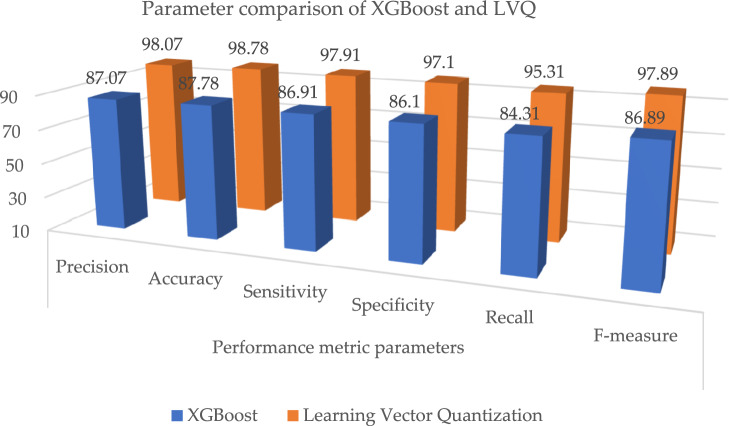
Graphical illustration of parameter metric comparison of XGBoost and LVQ.

**Figure 14:**
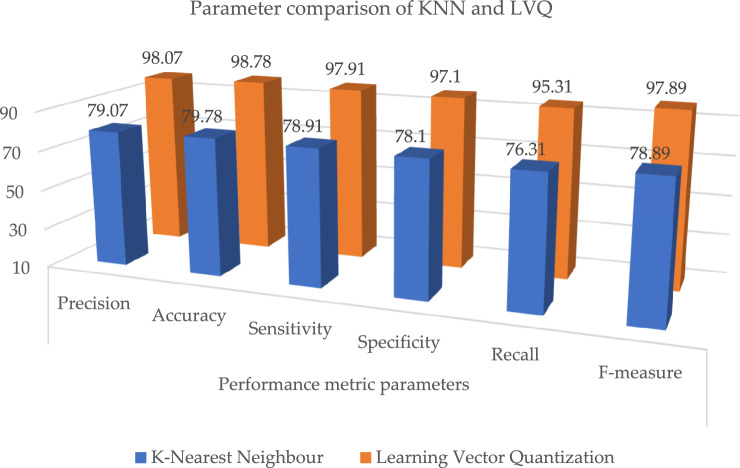
Graphical illustration of parameter metric comparison of KNN and LVQ.

The outcome shows that the LVQ obtained better outcome accuracy of 98.78%, and the graphical illustration is shown in Fig. 10. Tables 9 and 10, illustrates that the proposed system outcome is better than the XGBoost and KNN methods, and graphical view representation shown in Figs. 11 and 12 respectively. Table 11, depicts the paramenter metric (precision, accuracy, sensitivity, specificity, recall and f-measure) comparison outcome of support vector machine (DT) and learning vector quantization (LVQ). Then, Table 12, shows the performance metric parameter comparison of various classifiers such as, DT, KNN, RF, SVM and XGBoost. From the Table 12, the proposed system achieved (Pre 98.07%, Acc 98.78%, Se 97.91%, Sp 97.1%, Recall 95.31% and Fm 97.89%) better outcome in all parameters than the other conventional techniques. Figure 13, depicts the graphical illustration of parameter metric comparison of XGBoost and LVQ. Figure 14, represents the graphical illustration of parameter metric comparison of KNN and LVQ.

**Table 9:** Parameter metric comparison of XGBoost and LVQ.

Classification techniques	Performance metric parameters					
	Precision	Accuracy	Sensitivity	Specificity	Recall	F-measure
XGBoost	87.07	87.78	86.91	86.1	84.31	86.89
**Proposed Learning vector quantization**	**98.07**	**98.78**	**97.91**	**97.1**	**95.31**	**97.89**

Significant values are in bold.

**Table 10:** Parameter metric comparison of KNN and LVQ.

Classification techniques	Performance metric parameters					
	Precision	Accuracy	Sensitivity	Specificity	Recall	F-measure
K-Nearest Neighbour	79.07	79.78	78.91	78.1	76.31	78.89
Proposed learning vector quantization	**98.07**	**98.78**	**97.91**	**97.1**	**95.31**	**97.89**

Significant values are in bold.

**Table 11:** Parameter metric comparison of SVM and LVQ.

Classification techniques	Performance metric parameters					
	Precision	Accuracy	Sensitivity	Specificity	Recall	F-measure
Support vector machine	86.07	86.78	85.91	85.1	83.31	85.89
Proposed learning vector quantization	**98.07**	**98.78**	**97.91**	**97.1**	**95.31**	**97.89**

Significant values are in bold.

**Table 12:** Performance metric comparison of various classifiers.

Classification techniques	Performance metric parameters					
	Precision	Accuracy	Sensitivity	Specificity	Recall	F-measure
Random forest	88.07	88.78	87.91	87.1	85.31	87.89
Decision tree	89.07	89.78	88.91	88.1	86.31	88.89
Support vector machine	86.07	86.78	85.91	85.1	83.31	85.89
XGBoost	87.07	87.78	86.91	86.1	84.31	86.89
Radial basis functions	90.07	90.78	89.91	89.1	87.31	89.89
K-nearest neighbour	79.07	79.78	78.91	78.1	76.31	78.89
**Proposed learning vector quantization**	**98.07**	**98.78**	**97.91**	**97.1**	**95.31**	**97.89**
Naive Bayes	94.07	94.78	93.91	93.1	91.31	93.89

Significant values are in bold.

**Figure 15:**
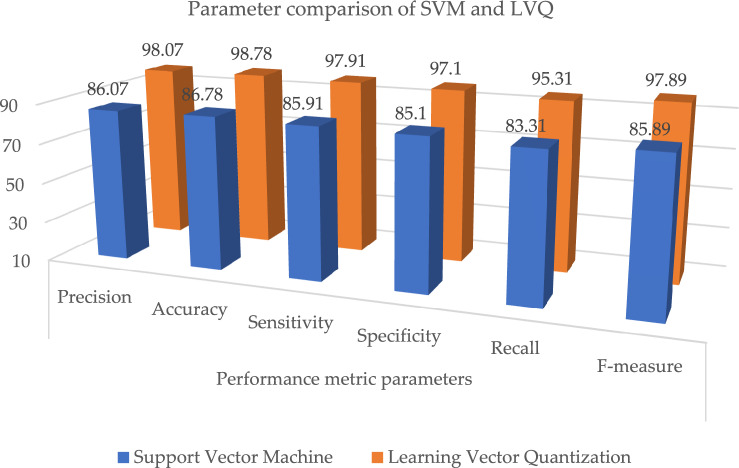
Graphical illustration of parameter metric comparison of SVM and LVQ.

**Figure 16:**
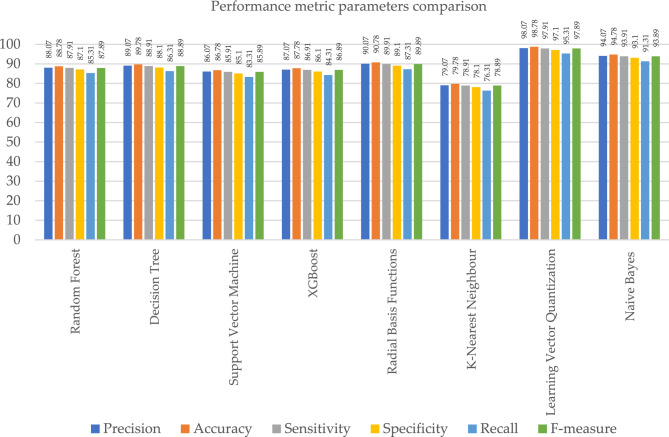
Classification accuracy—performance metric parameter comparison.

**Figure 17:**
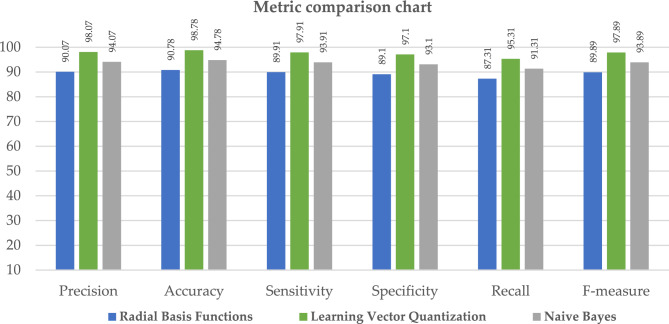
Performance metric comparison of RBF, NB and LVQ classifiers.

**Figure 18:**
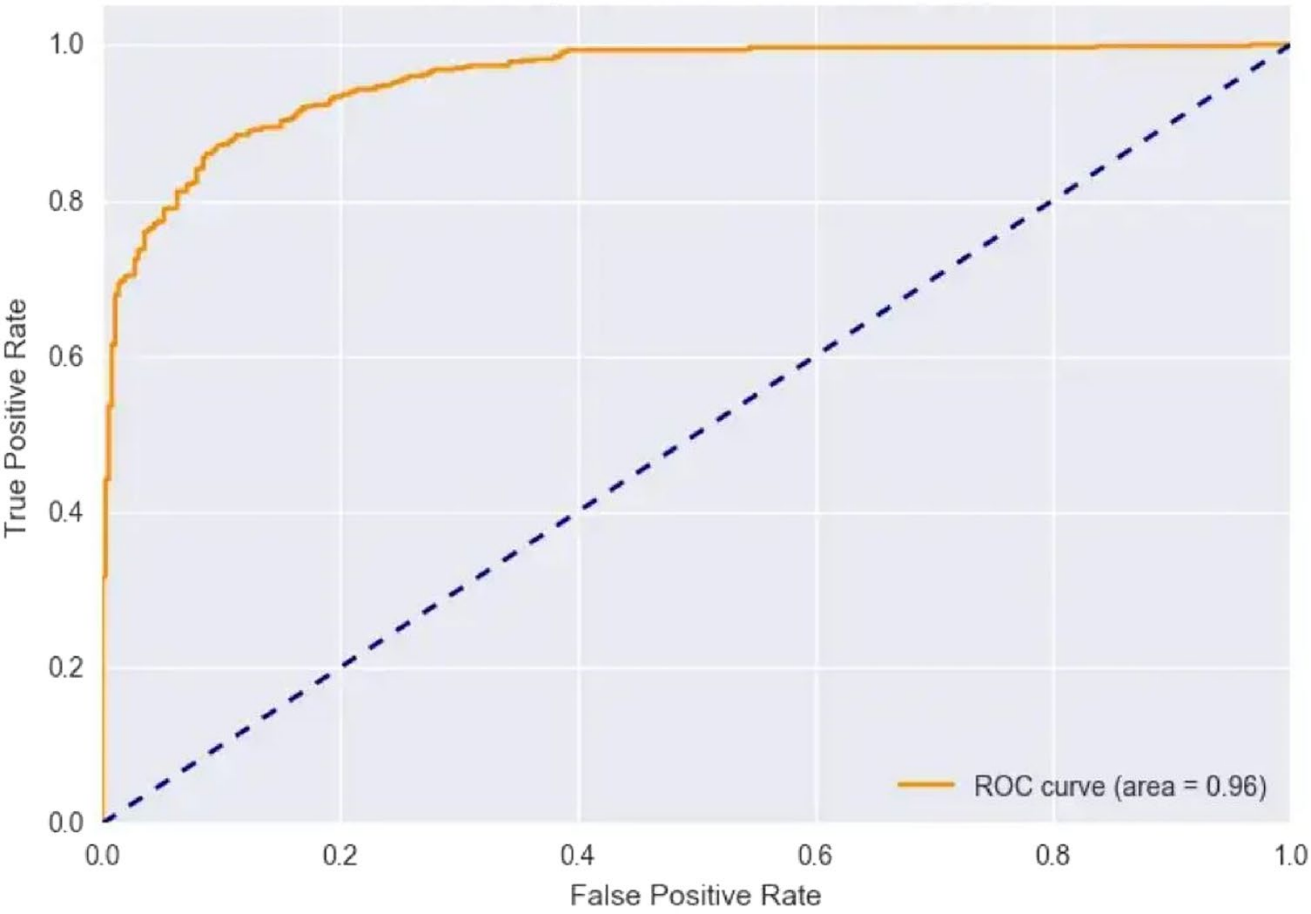
Learning vector quantization ROC.

When compared to the other classification techniques, the two corresponding techniques, radial basis function and nave Bayes, produced the best results. So, its respective parameters are taken, and it is compared with the proposed system shown in Table 13; the resultant shows that the proposed system parameter outcomes are better than those two outcomes, as illustrated in Fig. 15. Figure 16, depicts the proposed method performance metric parameter comparison of classification accuracy. Figure 17, illustrates the performance metric comparison of RBF, NB and LVQ classifiers. The receiver operating characteristics of the learning vector quantization are illustrated in Fig. 18.

**Table 13:** Parameters comparison for three respective classifiers.

Classification techniques	Performance metric parameters					
	Precision	Accuracy	Sensitivity	Specificity	Recall	F-measure
Radial basis functions	90.07	90.78	89.91	89.1	87.31	89.89
Naive Bayes	94.07	94.78	93.91	93.1	91.31	93.89
**Proposed learning vector quantization**	**98.07**	**98.78**	**97.91**	**97.1**	**95.31**	**97.89**

Significant values are in bold.

## Conclusion

In this study, machine learning classifiers are utilised to determine whether or not a patient has heart problems. The dataset was taken from the repository at UCI. Following data collection, they will go through cleaning and pre-processing steps. Following this step, machine learning models are used for predictive analysis. We investigated the potential of these eight applied machine learning methods for making accurate predictions about cardiac disease. The inclusion criteria for these algorithms are that they be mature, representative, and at the state of the art in their respective fields. We have previously used the Naive Bayes and RBF neural networks, but other scholars have not used them on the UCI cardiovascular disease dataset. As a result, we have achieved a higher level of accuracy than they have, as shown in the table titled "state of the art," which compares our results to those of other researchers. The final findings demonstrate that when the learning machine classifiers were put to use, the Naive Bayes and RBF neural networks achieved an accuracy of 94.78% when attempting to forecast the presence of coronary cardiovascular disease. However, the Learning Vector Quantization method achieved the highest categorization accuracy of 98.78%, with a specificity of 97.1% and sensitivity of 97.91%, a precision of 98.07% and 95.31%, and 97.89% F1score and F-measure values, respectively.

## Future work

In the future, our research aims to further enhance the reliability of our conclusions by incorporating additional datasets. We will explore the use of metaheuristic techniques and nature-inspired algorithms to optimize the parameters of machine learning classifiers and deep learning methods. This optimization process will enable us to more effectively evaluate the presence of heart disease across various heart disease-related datasets. Additionally, we will focus on improving the accuracy of existing algorithms to enhance their performance in detecting heart disease. By leveraging these advancements, we aim to provide more robust and accurate methods for the diagnosis and evaluation of heart disease.

## Data Availability

Used publicly available database, and no human data/sample used in the study” https://archive.ics.uci.edu/ml/datasets/heart+disease.
