# Author Correction: An active learning machine technique based prediction of cardiovascular heart disease from UCI-repository database

**DOI:** 10.1038/s41598-024-66981-3

**Published:** 2024-07-23

**Authors:** Saravanan Srinivasan, Subathra Gunasekaran, Sandeep Kumar Mathivanan, Benjula Anbu Malar M. B, Prabhu Jayagopal, Gemmachis Teshite Dalu

**Affiliations:** 1 Department of Computer Science and Engineering, Vel Tech Rangarajan Dr.Sagunthala R&D Institute of Science and Technology, Avadi, Chennai India; 2 Department of Computer Science and Engineering, Sathyabama Institute of Science and Technology, Chennai, India; 3 School of Computing Science and Engineering, Galgotias University, Greater Noida, 203201 India; 4 School of Computer Science Engineering and Information Systems, Vellore Institute of Technology, Vellore, 632014 Tamil Nadu India; 5 Department of Software Engineering, College of Computing and Informatics, Haramaya University, POB 138, Dire Dawa, Ethiopia

Correction to: *Scientific Reports* 10.1038/s41598-023-40717-1, published online 21 August 2023

In the original version of this Article, in the Proposed methodology section, under the subheading ‘Learning vector quantization—cardiovascular classification’, the explanation of the algorithm ‘LVQ’ was incomplete.

As a result,

“Step 1: Start.

Step 2: Reference vector initialization based on the training vectors and denotes ‘m’ is the cluster numbers and it can be used as a weight vector. Rest of the vectors will be assigned for training mode.

Step 3: Randomly assigning the initial classification and its corresponding weights.

Step 4: Initializing K-means clustering technique.

Step 5: The reference vector β is assigned.

Step 6: Computing the square of Euclidean distance for, i and j (1-to-m and 1-to-n) respectively.

$$ED(j)=\sum_{i=1}^{n}\sum_{j=1}^{m}{({\alpha }_{i}-{X}_{i,j})}^{2}$$

Step 7: To compute and achieve the raising unit J where ED is locally minimum.

Step 8: Compute the initial weight of the raising unit using the relative conditions,

$${X}_{j}^{new}={X}_{j}^{old}+\beta (\alpha -{X}_{j}^{old})$$

$${X}_{j}^{new}={X}_{j}^{old}-\beta (\alpha -{X}_{j}^{old})$$

Step 9: Lessen the β learning rate.

Step 10: Initiate the stopping condition of testing.

Step 11: Stop”

now reads:

“Algorithm 1: Learning vector quantization—cardiovascular classification

1. Clarification of parameters:
  m (number of reference vectors): This should be specified as a parameter that determines how many reference vectors (or prototype vectors) will be used in the LVQ algorithm.
  Reference Vector β: There seems to be a confusion here. In LVQ, the reference vectors are not denoted by β. The learning rate, often denoted by or η, is the scalar that is adjusted during training.
2. Step-by-Step Clarification of LVQ Algorithm:
  Step 1: Start
  Step 2: Initialize the reference vectors (prototypes). This involves selecting m vectors from the training set to serve as the initial reference vectors for each class.
  Step 3: Randomly assign initial classifications to the reference vectors if not done explicitly.
  Step 4: Assign the initial learning rate β (often denoted by or η).
  Step 5: Compute the squared Euclidean distance between each training vector and each reference vector :
  Step 6: Find the reference vector Rj that is closest to the input vector (i.e., has the minimum Euclidean distance).
  $$ED(j)=\sum_{i=1}^{n}\sum_{j=1}^{m}{({\alpha }_{i}-{X}_{i,j})}^{2}$$
  Step 7: Update the reference vector based on the classification of the input vector:
  If the input vector belongs to the same class as the reference vector Rj (denoted by S = Rj), update the reference vector away from the input vector:
  This rule helps the reference vector better represent its class by moving towards the input vector from the same class.
  If the input vector belongs to the different class than the reference vector Rj (denoted by S ≠ Rj), update the reference vector away from the input vector:
  This rule helps to increase the distinction between classes by moving the reference vector away from the input vector of a different class.
  Step 8: Decrease the learning rate β according to a predefined schedule. This could be a linear decay, exponential decay, or another method.
  $${X}_{j}^{new}={X}_{j}^{old}+\beta (\alpha -{X}_{j}^{old})$$
  $${X}_{j}^{new}={X}_{j}^{old}-\beta (\alpha -{X}_{j}^{old})$$
  Step 9: Check for stopping conditions. Common conditions include reaching a maximum number of iterations or when changes in the reference vectors become negligible.
  Step 10: Stop
3. Addressing Variables and Notation:
  Represents the components of the reference vectors.
  Represents a training vector.
  Represents the class label of the training vector.

Rj—Represents the j-th reference vector.

β—Represents the learning rate.”

The original Article has been corrected.

